# Pro-Apoptotic and Immunotherapeutic Effects of Carbon Nanotubes Functionalized with Recombinant Human Surfactant Protein D on Leukemic Cells

**DOI:** 10.3390/ijms221910445

**Published:** 2021-09-28

**Authors:** Haseeb A. Khan, Uday Kishore, Hamed M. Alsulami, Salman H. Alrokayan

**Affiliations:** 1 Department of Biochemistry, College of Science, King Saud University, Riyadh 11451, Saudi Arabia; halsulami@gmail.com (H.M.A.); salrokayan@ksu.edu.sa (S.H.A.); 2 Biosciences, College of Health, Medicine and Life Sciences, Brunel University London, Uxbridge UB8 3PH, UK; ukishore@hotmail.com

**Keywords:** carbon nanotubes, human SP-D, cancer cells, apoptosis, immunotherapy

## Abstract

Nanoparticles are efficient drug delivery vehicles for targeting specific organs as well as systemic therapy for a range of diseases, including cancer. However, their interaction with the immune system offers an intriguing challenge. Due to the unique physico-chemical properties, carbon nanotubes (CNTs) are considered as nanocarriers of considerable interest in cancer diagnosis and therapy. CNTs, as a promising nanomaterial, are capable of both detecting as well as delivering drugs or small therapeutic molecules to tumour cells. In this study, we coupled a recombinant fragment of human surfactant protein D (rfhSP-D) with carboxymethyl-cellulose (CMC) CNTs (CMC-CNT, 10–20 nm diameter) for augmenting their apoptotic and immunotherapeutic properties using two leukemic cell lines. The cell viability of AML14.3D10 or K562 cancer cell lines was reduced when cultured with CMC-mwCNT-coupled-rfhSP-D (CNT + rfhSP-D) at 24 h. Increased levels of caspase 3, 7 and cleaved caspase 9 in CNT + rfhSP-D treated AML14.3D10 and K562 cells suggested an involvement of an intrinsic pathway of apoptosis. CNT + rfhSP-D treated leukemic cells also showed higher mRNA expression of p53 and cell cycle inhibitors (p21 and p27). This suggested a likely reduction in cdc2-cyclin B1, causing G2/M cell cycle arrest and p53-dependent apoptosis in AML14.3D10 cells, while p53-independent mechanisms appeared to be in operation in K562 cells. We suggest that CNT + rfhSP-D has therapeutic potential in targeting leukemic cells, irrespective of their p53 status, and thus, it is worth setting up pre-clinical trials in animal models.

## 1. Introduction

The innate immune system plays a key role in the clearance of pathogens and synthetic compounds including nanoparticles [1,2]. Nanoparticles have numerous biomedical applications [3,4,5,6], which can serve as drug delivery carriers or vaccine adjuvants [7]. Among nanoparticles, carbon nanotubes (CNTs) have unique physico-chemical properties, and hence, they are amenable as therapeutic nanocarriers [8,9,10]. CNTs can be single-walled (SWCNTs) and multiple-walled (MWCNTs), depending on length, diameter, and structure, and the layers of single CNT the wall is composed of [11].

Human surfactant protein D (SP-D) is a humoral, pathogen pattern recognition molecule, which is found to be associated with pulmonary surfactant, as well as mucosal surfaces outside the lungs [12,13]. SP-D belongs to the collectin family, a collagen containing C-type (calcium-dependent) lectin [14]. The primary structure of SP-D comprises a cross-linking amino-terminal region, a triple-helical collagen region, a coiled-coil neck region, and a C-type lectin domain or carbohydrate recognition domain (CRD) as a trimeric unit [15,16]. SP-D can bind to various carbohydrate and/or charge patterns on the surface of pathogens and become involved in clearing them by recruiting phagocytic cells such as neutrophils and macrophages [15,16]. SP-D can also interact with a range of cancer cell lines (leukemic, lung, pancreatic, prostate, ovarian and breast). For example, a truncated form of recombinant human SP-D (rfhSP-D), composed of trimeric neck and C-type lectin domain, has been shown to interfere with tumour progression via apoptosis induction, invasion, and epithelial-to-mesenchymal transition [17,18,19,20,21,22]. These studies have thus suggested that SP-D has an immune surveillance role against tumors.

SP-D can associate with nanoparticles and modulate their uptake by macrophages [23,24]. SP-D can bind efficiently with oxidized (Ox) DWCNTs via their C-type lectin domain [2,25]. SP-D mediated enhancement of nanoparticle uptake by alveolar macrophages and dendritic cells in mice has been examined using polystyrene, carbon black and silica nanocarriers [23].

CNTs, when opsonized with rfhSP-D, can provoke a differential pro-inflammatory immune response [26]. Surface modifications of hydrophobic CNTs are used for their good dispersion via covalent or non-covalent surface coatings [27]. For instance, the dispersion of MWNTs via oxidation (Ox-CNT), or with carboxymethyl-cellulose (CMC-CNT), has been reported [27]. Soluble complement components, such as factor H and C1q, opsonize functionalized CNTs, suggesting that key innate immune molecules can bind CNTs and alter inflammatory response [27].

This study was aimed at examining the ability of CNT + rfhSP-D to induce apoptosis using an eosinophilic cell line, AML14.3D10 [28], and a chronic myelogenous leukemia cell line, K562, to assess if CNT + rfhSP-D nanomaterials are worth testing in animal models.

## 2. Results

### 2.1. CNT + rfhSP-D Treatment Reduces Cell Viability of AML14.3D10 and K562 Leukemic Cell Lines

**Figure 1:**
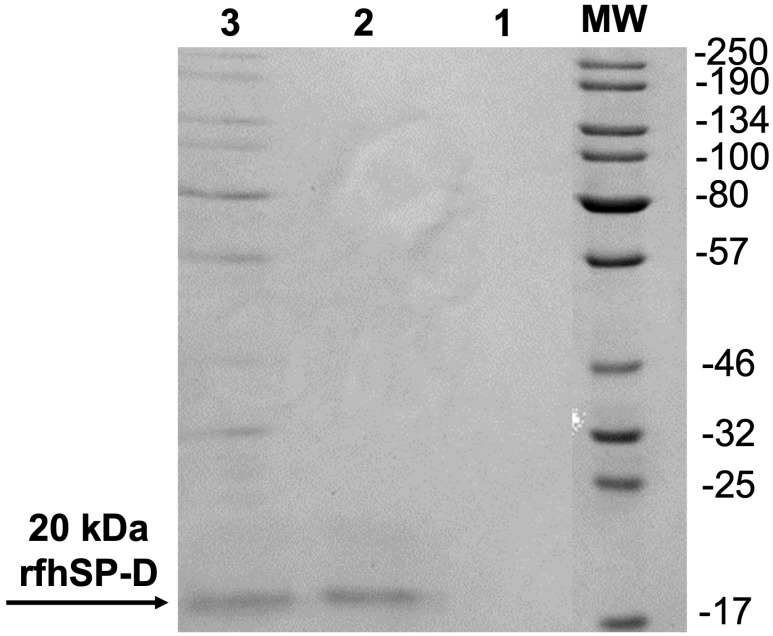
Purified rfhSP-D (10 µg/mL) or carboxymethyl cellulose-coated carbon nanotubes (rfh-SP-D-CNTs) coupled-rfhSP-D (10 µg/mL) samples were subjected to an SDS-PAGE (15% *v*/*v*). Lane 1: Supernatant (10 µL/well) taken after centrifugation of rfhSP-D-CNT. Lane 2: purified rfhSP-D. Lane 3: rfhSP-D-CNT. The original image is available as a Supplementary Materials.

**Figure 2:**
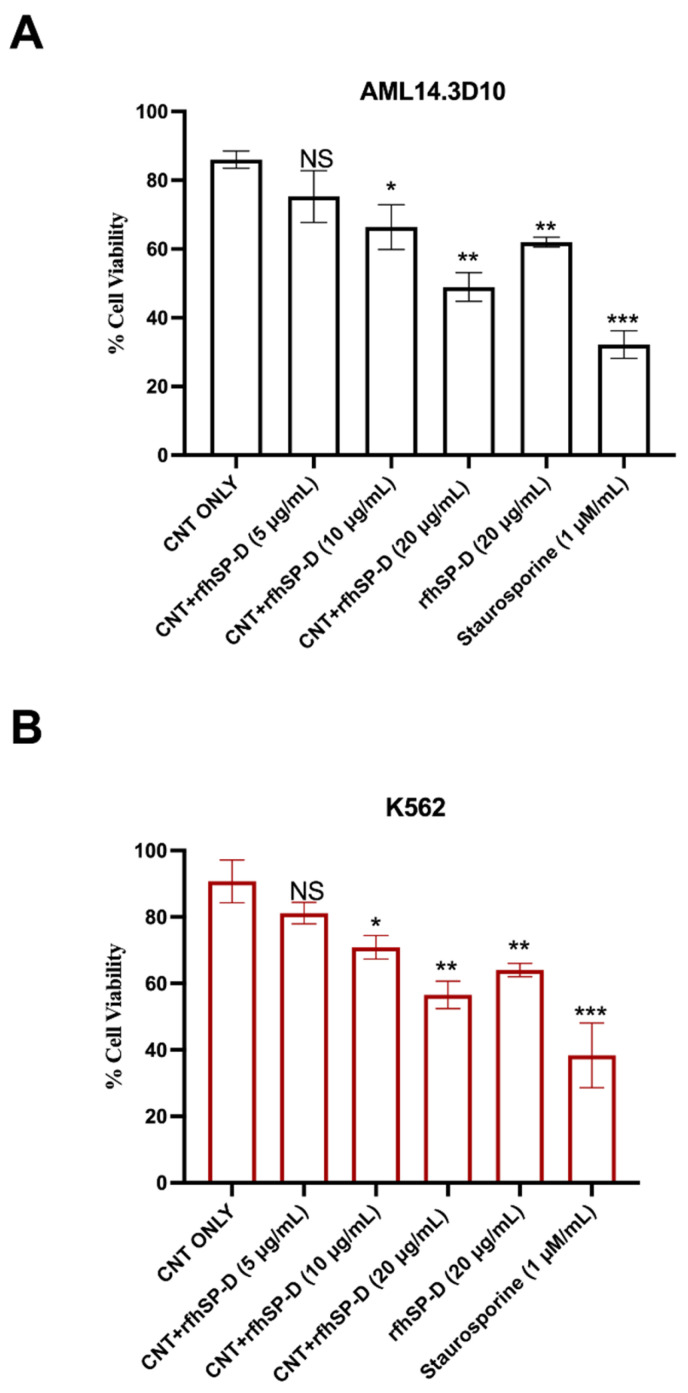
Cell viability following treatment with CNT + rfhSP-D-CNT in AML14.3D10 (**A**) and K562 (**B**) cell lines via trypan-blue-dye exclusion assay. Cells (0.1 × 10^5^) were treated with CNT + rfhSP-D (5, 10, 20 µg/mL), rfhSP-D (20 µg/mL) or CNT alone (20 µg/mL) for 24 h at 37 °C. The data has been normalized with cells only as 100% of the cell viability. * *p* < 0.05, ** *p* < 0.01 and *** *p* < 0.001 compared to CNT only group.

**Figure 3:**
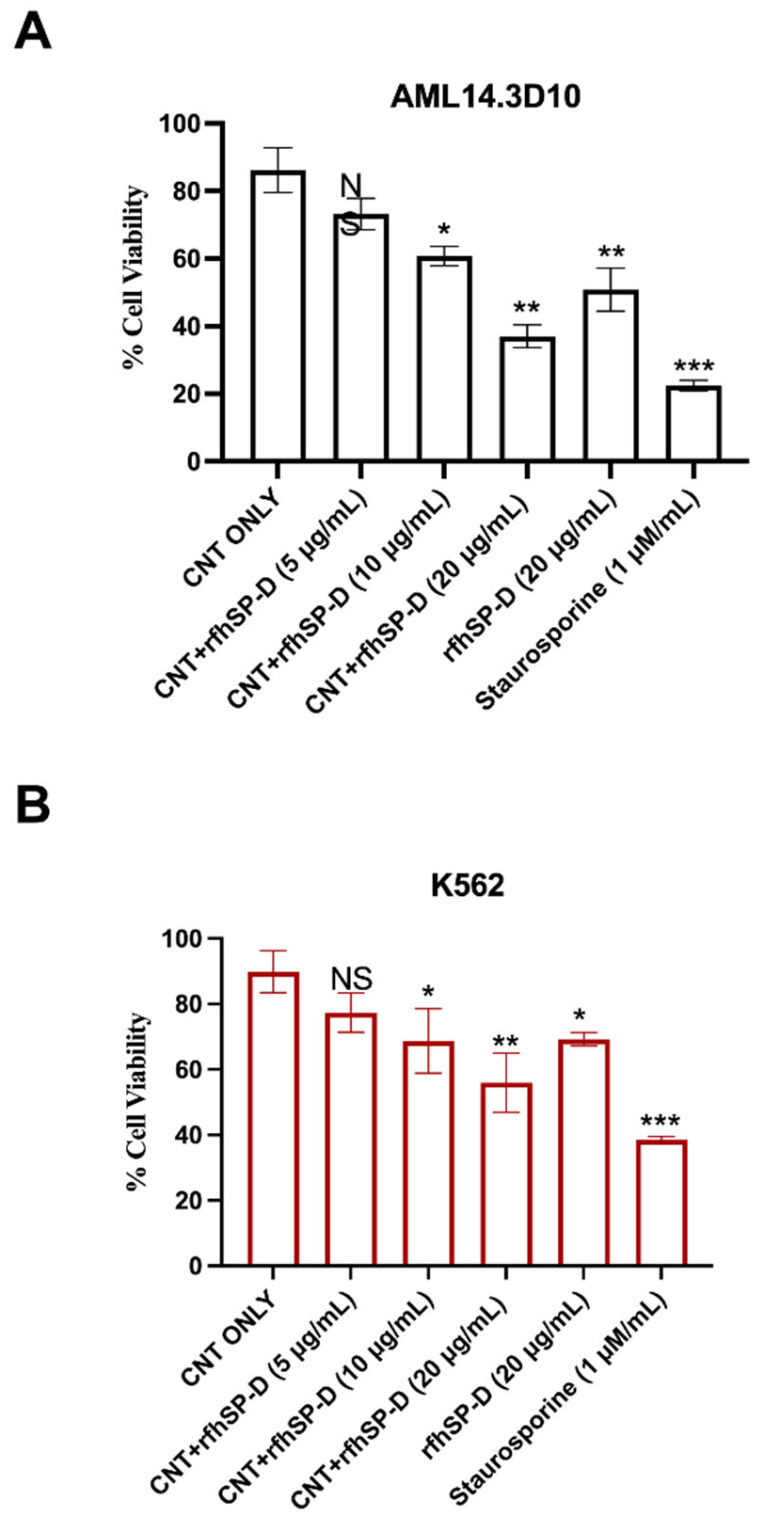
CNT + rfhSP-D treatment reduced viability of AML14.3D10 (**A**) and K562 (**B**) cells, as measured by MTT assay. The data have been normalized with cells only as 100% of the cell viability. Values are means ± SEM (*n* = 3) * *p* < 0.05, ** *p* < 0.01 and *** *p* < 0.001 compared to CNT only group.

First, we analysed and confirmed the stable binding of purified rfhSP-D with CMC-MWCNTs, as evident from the SDS-PAGE (Figure 1). Supernatant after centrifugation was also loaded, which did not show presence of rfhSP-D. rfhSP-D (10 µg/mL), without the addition of CNT, was used as a positive control. The quantitative analysis of viability in treated (cells + CNT + rfhSP-D; 5, 10, and 20 μg/mL in serum-free RPMI medium; cells + CNT as control) leukemic cells was carried out using trypan blue (Figure 2) and MTT (Figure 3) assays at 24 h time point. Trypan blue exclusion assay revealed a significant reduction in the cell viability in CNT + rfhSP-D treated cell lines (AML14.3D10: ~48%; K562: ~56%) at 24 h in a dose-dependent manner (Figure 2). This was confirmed by the MTT assay: AML14.3D10 (~37%) and K562 (~55%) (Figure 3). As evident by the MTT assay, rfhSP-D (20 µg/mL) alone was also able to reduce cell viability in both AML14.3D10 (~51%) and K562 (~69%) cell lines.

### 2.2. Proliferation of AML14.3D10 and K562 Cell Lines Is Reduced following CNT + rfhSP-D Treatment

**Figure 4:**
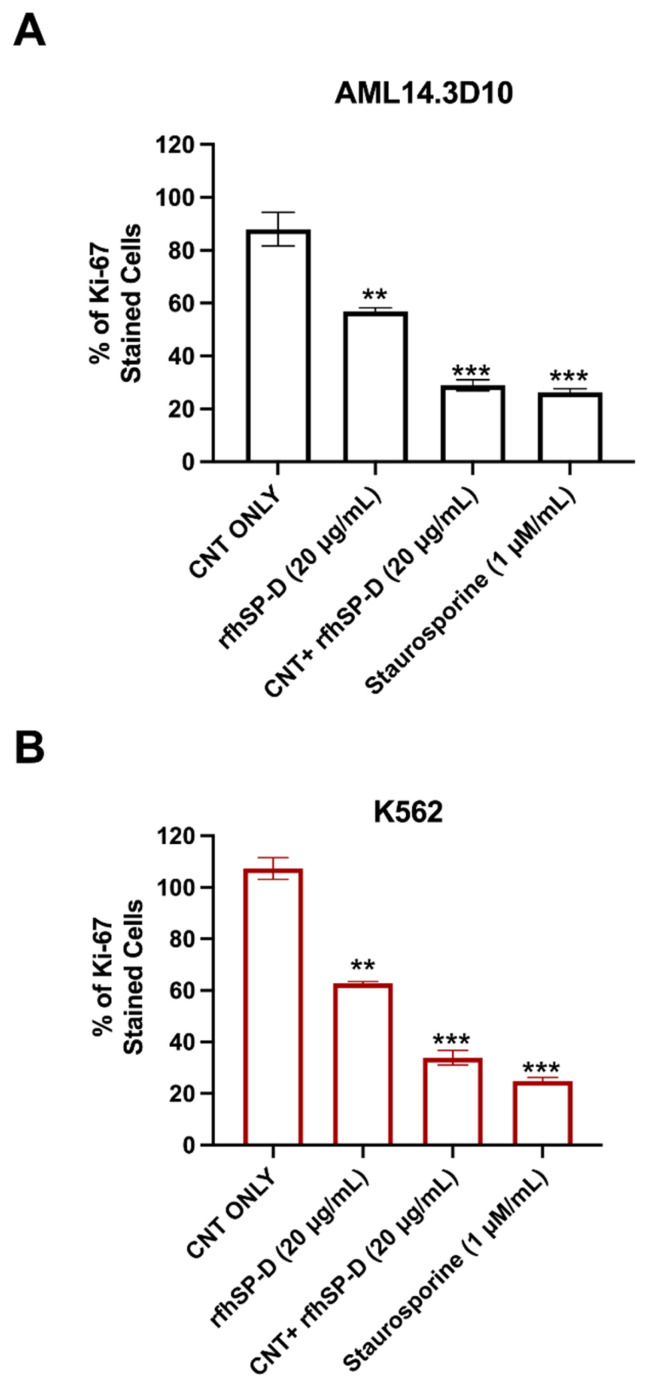
Anti-proliferative effects of CNT + rfhSP-D on AML14.3D10 (**A**) and K562 (**B**) cell lines. Values are means ± SD. ** *p* < 0.01, and *** *p* < 0.001 compared to CNT group only. The raw data are available as Supplementary Materials.

Experiments were carried out to determine whether CNT + rfhSP-D (20 µg/mL) affected AML14.3D10 and K562 cell proliferation (Figure 4). Mouse anti-Ki-67 antibody staining was used to measure the percentage proliferation. CNT + rfhSP-D treated AML14.3D10 cells showed ~29% cell proliferation compared to rfhSP-D alone (20 µg/mL) (~57%) (Figure 4). However, a higher percentage of cell proliferation was noted in CNT-treated AML14.3D10 cells (~88%). In the case of K562 cell line (Figure 4B), approximately ~34% cell proliferation was noticed following CNT + rfhSP-D treatment (compared to CNT alone; ~107% proliferative cells stained with Ki-67 antibody), suggesting that cells treated only with CNT continued to proliferate and grow unhindered. rfhSP-D alone (20 µg/mL) treatment was also capable of reducing proliferation of AML14.3D10 (~57%) and K562 (~63%) cells when compared to CNT alone. These data suggested that CNT + rfhSP-D was more effective in reducing cell proliferation of both AML14.3D10 and K562 cell lines, indicating its therapeutic potential against acute and chronic leukemic cell lines.

### 2.3. Apoptosis Induction by CNT + rfhSP-D in AML14.3D10 and K562 Cell Lines

**Figure 5:**
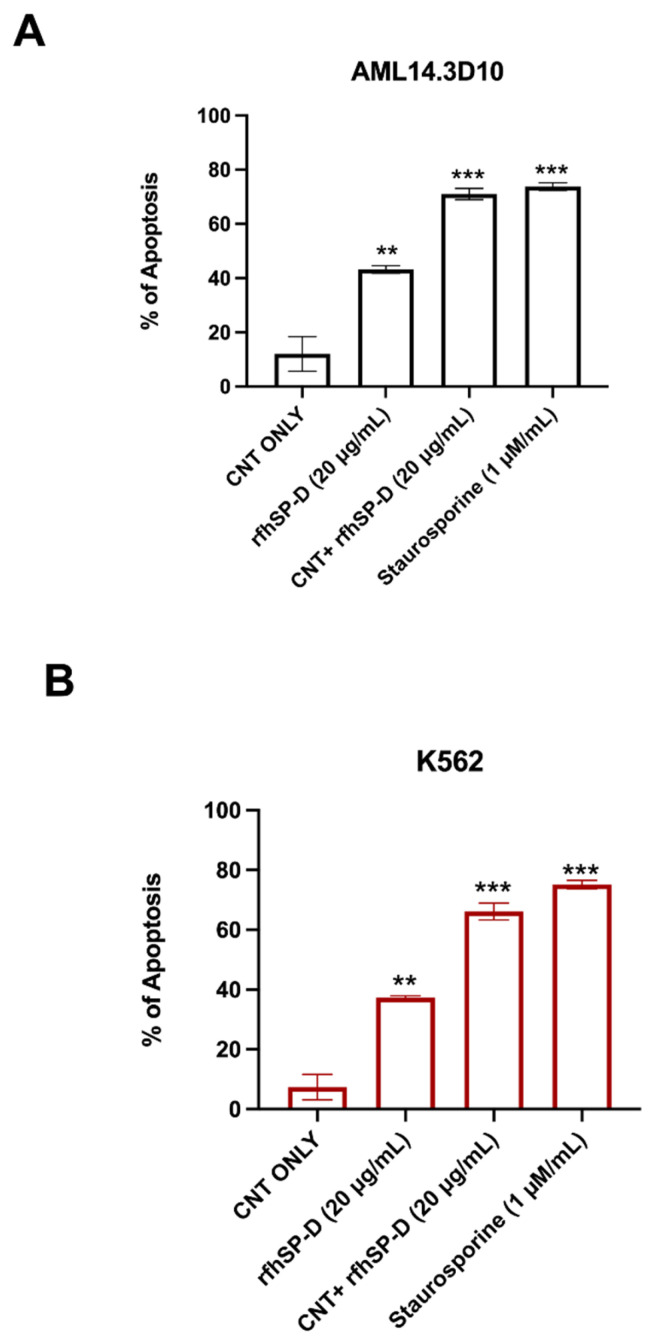
Flow cytometry analysis of apoptosis induction in AML14.3D10 (**A**) or K562 (**B**) cell lines treated with CNT + rfhSP-D. For Annexin V/FITC and DNA/PI staining, 12,000 cells were acquired and plotted. Values are means ± SEM (*n* = 3). ** *p* < 0.01 and *** *p* < 0.001 compared to CNT only group. The raw data are available in the Supplementary Materials.

The quantitative analysis of apoptosis induction by CNT + rfhSP-D was performed using flow cytometry. A significant proportion of AML14.3D10 or K562 (Figure 5) cells treated with CNT + rfhSP-D (20 µg/mL), or rfhSP-D (20 µg/mL) alone, resulted in increased apoptosis induction at 24 h, compared to CNT alone (untreated control). CNT + rfhSP-D was effective in inducing the maximum apoptosis at 24 h; AML14.3D10 (~71%) and K562 (~66%), when compared to CNT alone [AML14.3D10 (~12%) and K562 (~7%)]. rfhSP-D (20 µg/mL) alone was also able to reduce cell viability in both AML14.3D10 (~43%) (Figure 5) and K562 (~37%) cell lines (Figure 5; Supplementary Materials). This assay is based on the ability of annexin V/FITC to bind to phosphatidylserine (PS) on apoptosing cells. A higher percentage of PI positive AML14.3D10 compared to K562 cells appeared to suggest that these cells were late apoptotic/necrotic. Staurosporine (1 μM/mL), used as a positive control for triggering apoptosis, brought about ~72% apoptosis at 24 h.

### 2.4. Up-Regulation of Cell-Cycle Inhibitors by CNT + rfhSP-D Treatment

**Figure 6:**
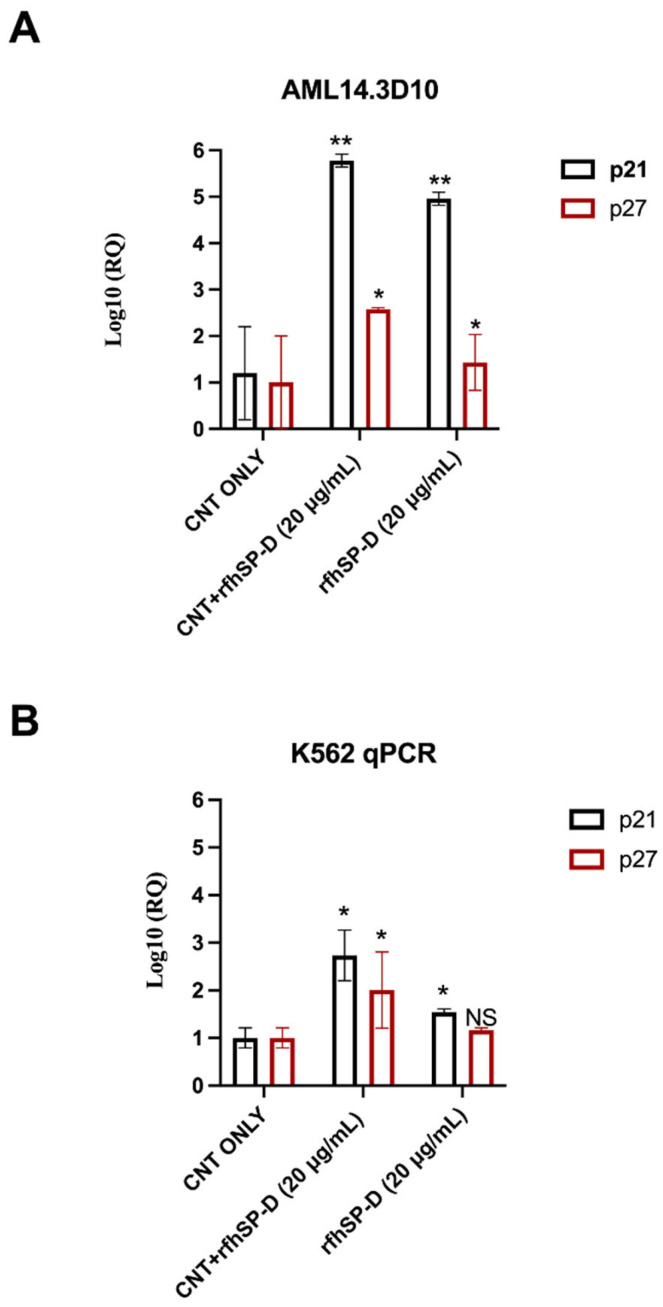
CNT + rfhSP-D treatment causes the upregulation of p21 and p27 cell cycle inhibitors in AML14.3D10 (**A**) and K562 (**B**) cell lines. AML14.3D10 or K562 (0.4 × 10^6^) cells, treated with CNT + rfhSP-D (20 µg/mL) or rfhSP-D (20 µg/mL), plus untreated control (cells + CNT) (20 µg/mL), were used for RNA extraction, cDNA synthesis and RT-qPCR, using 18S as an endogenous control. The RQ value was calculated using the formula: RQ = 2 − ΔΔCt. Values represent means ± SEM (*n* = 3). * *p* < 0.05 and ** *p* < 0.01 compared to CNT only group.

To further understand the mechanism of apoptosis induced by CNT + rfhSP-D in AML14.3D10 or K562 cells, we analysed the expression of cell cycle inhibitors by qRT-PCR. p21 was upregulated in CNT + rfhSP-D treated AML14.3D10 (log_10_ 5.7-fold) and K562 (log_10_ 2.7-fold) (Figure 6) [compared to CNT alone: AML14.3D10 (log_10_ 1.2-fold) and K562 (log_10_ 1-fold)]. p27 transcripts were also upregulated in CNT + rfhSP-D challenged AML14.3D10 (log_10_ 2.5-fold) and K562 (log_10_ 2-fold) cells. The level of upregulation was considerably higher compared to CNT or rfhSP-D alone that were negative and positive controls, respectively, suggesting that coating rfhSP-D on CNTs enhanced rfhSP-D potency for targeting tumors.

### 2.5. rfhSP-D Upregulates p53 Expression in AML14.3D10 Cell Line

**Figure 7:**
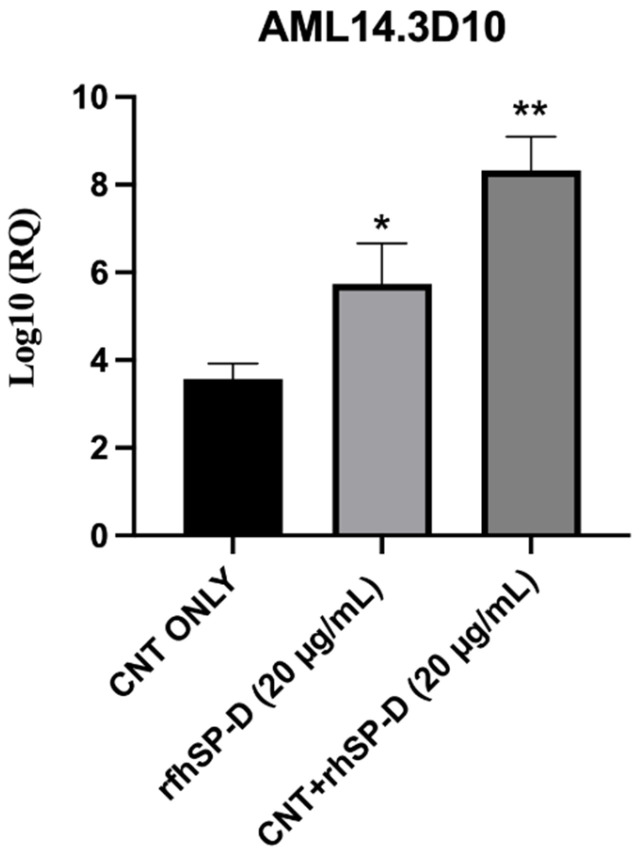
CNT + rfhSP-D treated AML14.3D10 cells show upregulation of the mRNA transcript levels of p53. AML14.3D10 (0.4 × 10^6^) cells were treated with CNT + rfhSP-D or rfhSP-D alone, along with an untreated control (cells + CNT) (20 µg/mL each). The RQ value was calculated using the formula: RQ = 2 − ΔΔCt. * *p* < 0.05 and ** *p* < 0.01 compared to CNT only group.

p53, a transcription factor, regulates oncogenic responses including DNA damage, cell cycle arrest, and apoptosis. CNT + rfhSP-D or rfhSP-D alone treated AML14.3D10 cells showed increased transcript levels of p53 when compared to untreated cells. CNT + rfhSP-D treated cells showed log_10_ 8.2-fold increased mRNA levels, compared to rfhSP-D treated cells (approximately log_10_ 5.2-fold) (Figure 7). p53 transcripts were not measured in K562 cells as these cells do not express wild type p53. These data suggest that CNT + rfhSP-D treatment can induce apoptosis in these cell lines regardless of their p53 status.

### 2.6. Apoptosis Induction in AML14.3D10 and K562 Cells by rfhSP-D-CNT via Intrinsic Pathway

**Figure 8:**
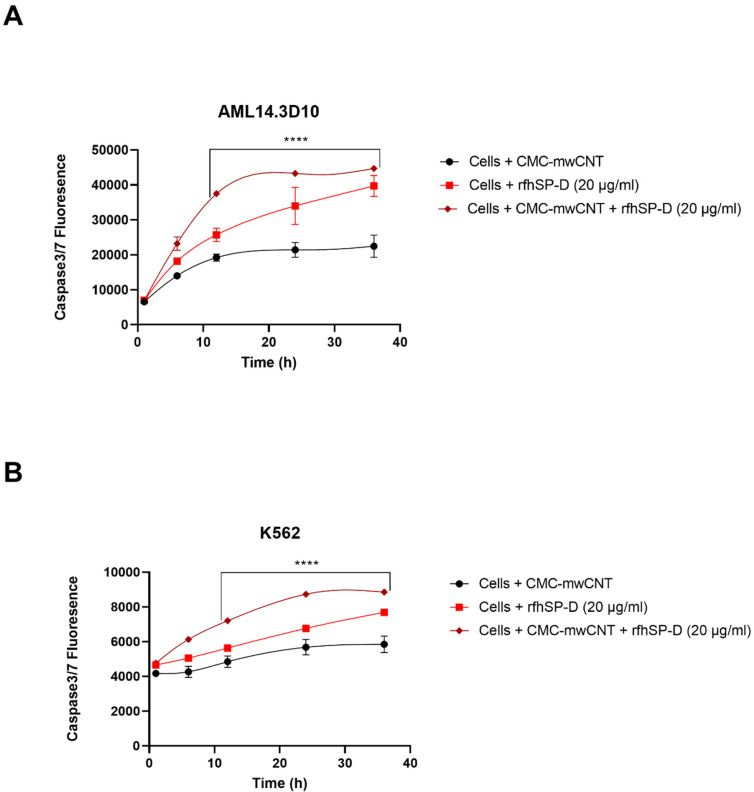
Activation of caspase 3/7 in AML14.3D10 (**A**) or K562 (**B**) cell lines following CNT + rfhSP-D treatment. AML14.3D10 or K562 cells (0.1 × 10^5^) were seeded and challenged with CNT + rfhSP-D (20 µg/mL) or rfhSP-D (20 µg/mL) Cells + CNT was used as an untreated control. **** *p* < 0.0001 versus control group (*n* = 3).

**Figure 9:**
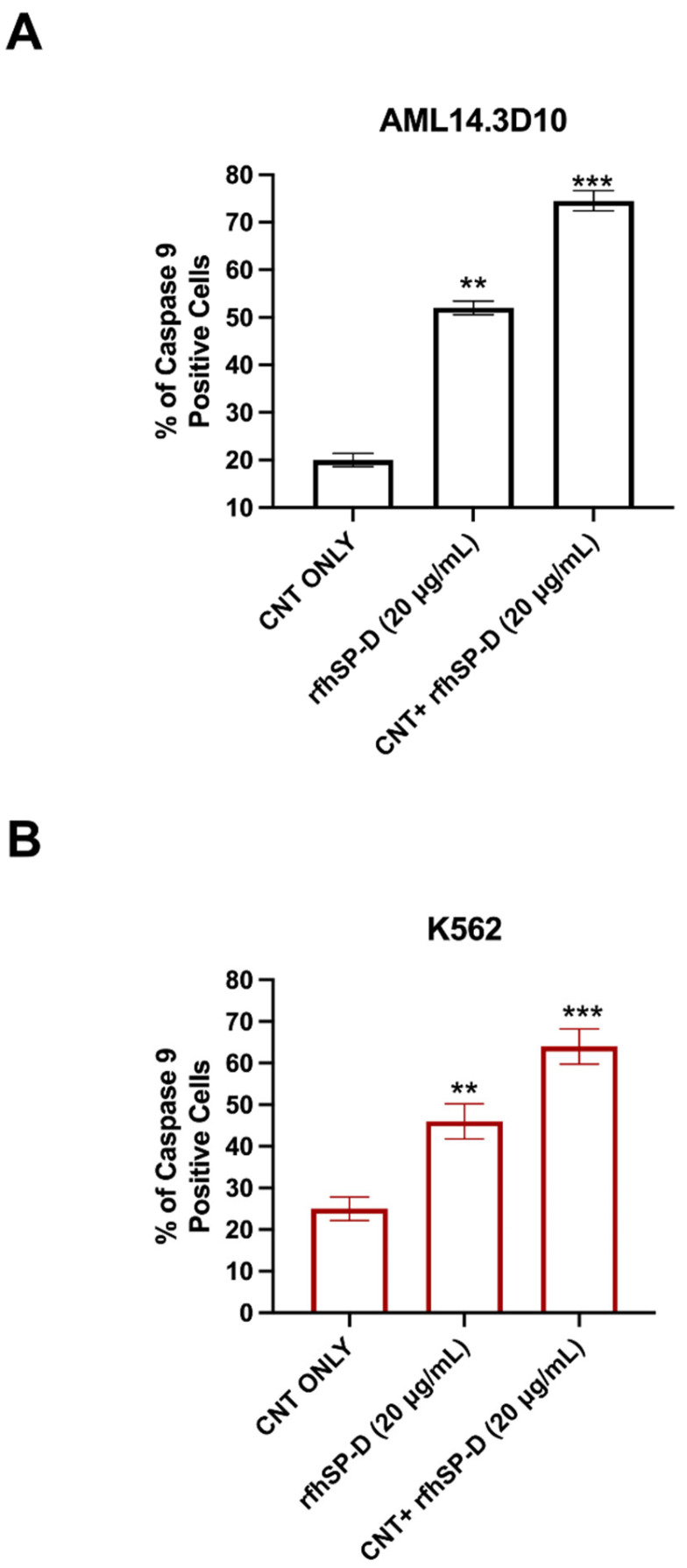
CNT + rfhSP-D treatment upregulates the levels of cleaved caspase 9 on AML14.3D10 (**A**) or K562 (**B**) cell lines at 24 h. AML14.3D10 or K562 cells (0.4 × 10^6^) were treated with rfhSP-CNT or rfhSP-D, along with an untreated control (cells + CNT). Values are expressed as mean ± SD (*n* = 3). ** *p* < 0.01, and *** *p* < 0.001 versus control group.

Since apoptosis can be initiated via intrinsic or extrinsic pathways, expression of caspases was examined in AML14.3D10 or K562 cell lines treated with CNT + rfhSP-D (20 μg/mL) or rfhSP-D alone (20 μg/mL), using a fluorogenic substrate to detect the activation of caspase 3 and 7 (Figure 8). Higher levels of caspase 3 and 7 were observed in CNT + rfhSP-D treated AML14.3D10 (Figure 8A) and K562 (Figure 8B) cells, when compared to rfhSP-D or CNT alone-treated cells. There was a time-dependent increase in caspase 3 and 7 activation, which peaked at 24 h. Cleaved caspase 9 level was observed in CNT + rfhSP-D (or rfhSP-D-treated) AML14.3D10 or K562 cells at 12h, reflecting an intrinsic pathway (Figure 9).

## 3. Discussion

The involvement of innate immune mechanisms in cancer progression and resistance has opened up opportunities for using innate immune molecules as a part of anti-tumour therapeutic strategies. Immune system, innate as well as adaptive, is a double-edged sword that can either foster tumour progression via immunosuppression, angiogenesis, and metastasis, or resist oncogenesis [29,30]. SP-D, especially the trimeric CRDs in its recombinant form (rfhSP-D), has recently been shown to be protective against a range of cancer, based on in vitro studies. Coupling rfhSP-D with nanoparticles triggers a differential immune response [26]. rfhSP-D-bound CNTs upregulate the pro-inflammatory response (IL-1β, TNF-α, IL-6 and IL-12) in U937 and THP-1 cells [26]. Here, we examined the ability of CNT + rfhSP-D to act as a potent inducer of apoptosis in leukemic AML14.3D10 or K562 cell lines. CNT + rfhSP-D treatment reduced the cell viability of AML14.3D10 and K562 cells and induced apoptosis at 24 h in a dose- and time-dependent manner, peaking at 24 h and 20 μg/mL. A significant reduction in viability was observed in CNT + rfhSP-D treated AML14.3D10 (~37%) and K562 (~55%) cells compared to untreated control (cells + CNT), based on trypan blue and MTT assays.

FACS analysis revealed a significant increase in the percentage of Annexin V-/PI-positive leukemic cells following CNT + rfhSP-D treatment, characterized by the disruption of the asymmetric arrangement of the membrane, and appearance of PS on the outer side of the cell membrane in the cells undergoing apoptosis [31]. Annexin V, a 36 kDa protein, can bind PS, and also enter the entire plasma membrane in necrotic cells. CNT + rfhSP-D triggered the maximum apoptosis at 24 h [AML14.3D10 (~71%) and K562 (~66%)], when compared to CNT alone [AML14.3D10 (~12%) and K562 (~7%)]. However, no significant difference in terms of cell viability reduction/apoptosis induction was noticed following rfhSP-D treatment at 48 h in AML14.3D10 and K562 cells, suggesting recovery of the cells after 24 h. Apoptosis induction in AML14.3D10 and K562 cell lines by CNT + rfhSP-D may occur through the intrinsic pathway, supported by increased levels of caspase 3, 7 and cleaved caspase 9. This validates earlier studies on AML14.3D10, prostate and breast cancer cells [20,21,32], and the involvement of a mitochondrial pathway [20,21,32].

We also tried to understand the underlying mechanism of apoptosis induction by CNT + rfhSP-D and the associated signaling pathways. CNT + rfhSP-D caused increased transcript level of p53 in AML14.3D10 cell line, probably due to oxidative stress [17,33]. The upregulation of p53 in CNT + rfhSP-D treated AML14.3D10 cells may downregulate pAkt pathway, increasing Bad and Bax, which in turn, causes the release of the cytochrome c, and caspase 9 cleavage. In addition, the increased expression of p53 and cell cycle inhibitors (p21/p27) can cause inactivation of the cyclin B–cdc2 complex, crucial for G2/M cell cycle transition [17]. The existence of a lack of p53 wild type gene in K562 cell line, and its increased susceptibility to CNT + rfhSP-D, the protective effects of rfhSP-D bound to CNTs seem p53 independent. An involvement of cellular receptors expressed by these cancer cell lines is of paramount importance. SP-D interaction with HMGA1, CD14, CD91-calreticulin complex, SIRPα, EGFR, and GRP78 has been reported [20,21,22,33,34]. The presence of rfhSP-D on CNT as an array of therapeutic molecule is likely to have a clustering effect on these putative receptors, enhancing the potency of rfhSP-D.

In conclusion, CNT + rfhSP-D nanomaterial seems to be an attractive and novel therapeutic approach for targeting intracellular signaling cascades. There is a clear therapeutic potential of rfhSP-D against tumour cells. The advantage here is that the enhanced glycosylation of oncogenic targets can evade natural or therapeutic antibodies. Having established the specific nature of interactions between CNT + rfhSP-D and receptors found on leukemic cancer cells, we can hope to investigate host response in the murine models of cancer using wild type and SP-D knock-out mice.

## 4. Materials and Methods

### 4.1. Cell Culture

AML14.3D10 and K562 cells (ATCC) were cultured in RMPI media containing 10% *v*/*v* fetal calf serum (FCS), 2 mM L-glutamine, and penicillin (100 U/mL)/streptomycin (100 µg/mL) (ThermoFisher Scientific, Oxford, UK). Cells were grown at 37 °C under 5% *v*/*v* CO_2_ until 80–90% confluency was reached.

### 4.2. Dispersion and Functionalization of CNTs

The CNTs used in this study were characterized and functionalized as previously described [26,27]. Briefly, CNTs (diameters 10–20 nm, length 5–20 µm; Arry Nano) were dispersed using CNT sulfuric acid/nitric acid (3:1 ratio) via sonication and functionalized using CMC (Sigma-Aldrich/Merck, Dorset, UK) in a 1:2 mass ratio [26,27].

### 4.3. Expression and Purification of rfhSP-D

A recombinant fragment of human SP-D (rfhSP-D) was expressed and purified as described previously [17,32]. Affinity purified rfhSP-D was then subjected to endotoxin level measurement using QCL-1000 Limulus amebocyte lysate system (Lonza, Slough, UK); the endotoxin levels were found to be ~5 pg/μg of rfhSP-D.

### 4.4. Sodium Dodecyl Sulfate Polyacrylamide Gel Electrophoresis (SDS-PAGE)

The binding of rfhSP-D to CMC-CNTs was assessed via SDS-PAGE (12% *v*/*v*). CNT + rfhSP-D samples were boiled in a treatment buffer containing SDS and β-mercaptoethanol at 95 °C for 10 min before loading on to the gel. The SDS-PAGE gel was stained for 2 h using brilliant blue stain containing methanol (50% *v*/*v*) and acetic acid (10% *v*/*v*). This followed submersion of the stained gel with gentle shaking with de-staining solution (staining solution without brilliant blue).

### 4.5. Trypan-Blue-Dye Exclusion Assay

AML14.3D10 or K562 cells (0.1 × 10^6^) were seeded in a 12-well plate in complete RPMI complete medium overnight under 5% CO_2_ at 37 °C. Next, the cells were washed with PBS and treated with CNT + rfhSP-D (5, 10 or 20 µg/mL), or rfhSP-D alone (20 µg/mL), in serum-free RPMI for 24 h. Cells + CNT and Staurosporine (1 µM/mL) were used as an untreated/negative and positive control, respectively. Cells were then washed, detached using 5 mM EDTA, and centrifuged (1200× *g*). The cell pellet, re-suspended in RPMI, was treated with Trypan blue (10 µL) (60%), and viable cells were counted using hemocytometer in 5 different optical fields with a threshold value of 200 cells per field.

### 4.6. MTT Assay

MTT [3-(4,5-dimethylthiazol-2-yl)-2,5-diphenyltetrazolium bromide] (Sigma-Aldrich, Dorset, UK) assay was performed to assess the cell metabolic activity (cells + CNT + rfhSP-D; cells + CNT). AML14.3D10 or K562 cells (0.1 × 10^5^) were seeded in 96-well plates in RPMI complete medium until 85% confluency, and treated with CNT + rfhSP-D (5, 10 or 20 µg/mL), or rfhSP-D (20 µg/mL), in serum free RPMI medium for 24 h. MTT (50 µg/µL) per well was added for 4 h at 37 °C. 25 µL medium per well was then mixed with 50 µL DMSO (10′, 37 °C), and the absorbance was read at 570 nm using an ELISA plate reader.

### 4.7. Flow Cytometry

For apoptosis assays, AML14.3D10 or K562 cells (0.4 × 10^6^) were seeded in culture petri dishes (Nunc) in complete RPMI medium for 24 h and treated with CNT + rfhSP-D (20 µg/mL), or rfhSP-D (20 µg/mL), in serum-free RPMI medium for 24 h. Other controls were used as described above. Detached, centrifuged and PBS washed cells were incubated with Alexa Fluor 488 (1:200) (Sigma-Aldrich/Merck, Dorset, UK) (15°, RT) in dark, and the extent of apoptosis was measured using Novocyte Flow Cytometer. Compensation parameters were acquired using unstained, untreated FITC stained, and untreated PI-stained samples for all the cell lines.

For proliferative studies, AML14.3D10 or K562 cells (0.4 × 10^6^) were washed with PBS, probed with anti-mouse Ki-67 (BioLegend, San Diego, CA, USA) diluted in permeabilization reagent of the FIX&PERM kit (Fisher Scientific), and incubated for 30 min at room temperature (RT). Goat anti-mouse-FITC conjugate (1:200) (Fisher Scientific) was used as a probe at RT in the dark for 30 min. Cells (12,000) were acquired for each experiment and compensated before plotting the acquired data.

For caspase 9 and 8 studies, AML14.3D10 or K562 cells (0.4 × 10^6^) were treated with CNT + rfhSP-D or rfhSP-D (cells + CNT as a control) for 24 h at 37 °C, and probed with rabbit anti-human cleaved caspase 9 or 8 (Cell Signaling Technology, Danvers, MA, USA) (1:200) for 1 h at RT. Cells were washed in PBS, incubated with Alexa Fluor 488 (1:200) (Sigma-Aldrich) at RT in dark for 30 min, acquired and compensated (12,000) prior to plotting the data.

### 4.8. Caspase-3/7 Analysis

AML14.3D10 or K562 cells (0.1 × 10^5^) were seeded in 96 well plates in RPMI complete medium until 80% confluency. The cells were then treated with CNTs, as described above, in serum-free RPMI medium containing CellEvent^TM^ Caspase-3/7 Green Detection Reagent (5 µM; Thermo-Fisher) (0, 10, 20, 30 or 40 h). Cells + CNT was used as an untreated/negative control. CellEvent^TM^ Caspase-3/7 Green Detection Reagent is a fluorogenic substrate for activated caspases 3 and 7 in cells undergoing apoptosis. The plates with treated and untreated samples were incubated at 37 °C with 5% CO_2_ to detect the levels of Caspase 3/7 using a Clariostar plus microplate reader (BMG Labtech, Cary, NC, USA).

### 4.9. Quantitative RT-PCR

AML14.3D10 or K562 cells (0.5 × 10^6^) were incubated with CNT + rfhSP-D (20 µg/mL) or rfhSP-D (20 µg/m in serum-free RPMI medium for 18 h and RNA was isolated using GenElute Mammalian Total RNA Purification Kit (Sigma-Aldrich) and treated with DNase I. 2 µg of total RNA was used for cDNA synthesis using High Capacity kit (Applied Biosystems/ThermoFisher, Abingdon, UK). Primer sequences were designed using Primer-BLAST software (Table 1). Each PCR reaction, carried out in triplicates, contained SYBR Green (5 µL) MasterMix (Applied Biosystems), primers (75 nM), and cDNA (500 ng) (7900HT; Applied Biosystems). The cycle involved 2′/50 °C and 10′/95 °C, and 40 cycles (15 s/95 °C; 1′/60 °C). Human 18S rRNA was used as a housekeeping gene control. Relative quantification (RQ) value and formula: RQ  =  2 − ΔΔCt were used to calculate the relative expression of each target. Cells + CNT was used as an untreated/negative control.

### 4.10. Statistical Analysis

The graphs were generated using the GraphPad Prism 6.0 software. A one-way ANOVA test was carried out for statistical significance analysis. values less than 0.05 were considered as statistically significant.

## Figures and Tables

**Table 1:** Target genes and terminal primers used in the RT-qPCR analysis.

Target Gene	Forward Primer	Reverse Primer
18S	5′-ATGGCCGTTCTTAGTTGGTG-3′	5′-CGCTGAGCCAGTCAGTGTAG-3′
P53	5′-AGCACTGTCCAACAACACCA-3′	5′-CTTCAGGTGGCTGGAGTGAG-3′
p21	5′-TGGAGACTCTCAGGGTCGAAA-3′	5′-CGGCGTTTGGAGTGGTAGAA-3′
p27	5′-CCGGTGGACCACGAAGAGT-3′	5′-GCTCGCCTCTTCCATGTCTC-3′

## Data Availability

Not applicable.

## Supplementary Materials

The following are available online at https://www.mdpi.com/article/10.3390/ijms221910445/s1.
