# Supplementary figures and images for: Pro-Apoptotic and Immunotherapeutic Effects of Carbon Nanotubes Functionalized with Recombinant Human Surfactant Protein D on Leukemic Cells
Source: Int J Mol Sci. 2021 Sep 28;22(19):10445. doi: 10.3390/ijms221910445 (PMC8508673; doi:10.3390/ijms221910445)

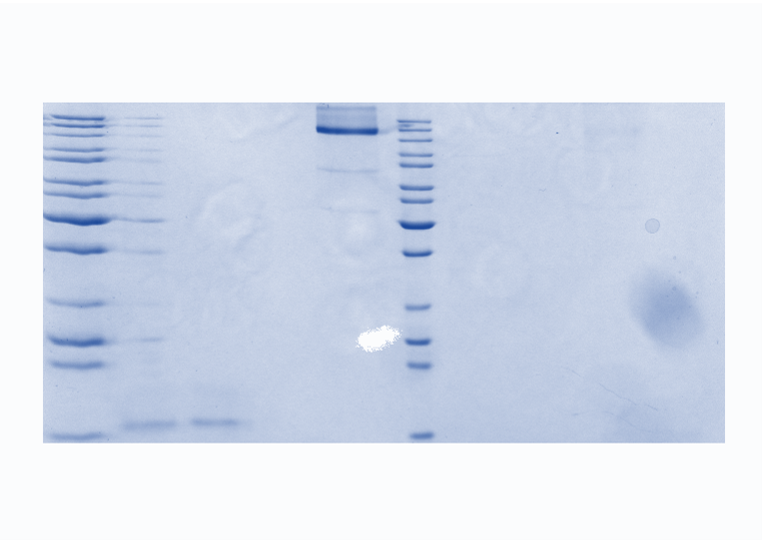

Supplement: Supplementary file 1 [file ijms-22-10445-s001.zip › ijms-1344399-supplementary.tif]
